# Neutrophilic Urticarial Dermatosis in Systemic Lupus Erythematosus: A Diagnostic Challenge

**DOI:** 10.7759/cureus.91851

**Published:** 2025-09-08

**Authors:** Udokama Ezekwe, Daphne Thampy, Jordan T Hyde, Sylvia Hsu

**Affiliations:** 1 Dermatology, Temple University Hospital, Philadelphia, USA; 2 Dermatopathology, Thomas Jefferson University Hospital, Philadelphia, USA

**Keywords:** autoimmune skin disorders, dermatopathology, neutrophilic urticarial dermatosis, systemic lupus erythematosus, urticarial vasculitis

## Abstract

Neutrophilic urticarial dermatosis (NUD) is a rare condition that clinically resembles urticaria but is distinguished histopathologically. Given the overlap of clinical and histopathologic features between NUD, urticaria, and urticarial vasculitis (UV), distinguishing between these diagnoses is crucial, as their treatments differ significantly.

A 47-year-old woman with systemic lupus erythematosus (SLE) presented with a mildly pruritic, burning rash for one week. Physical examination revealed erythematous, edematous wheals on her trunk and proximal extremities, indistinguishable from urticaria. A skin biopsy revealed a moderately dense infiltrate of neutrophils and nuclear dust with intact vessels, consistent with neutrophilic urticarial dermatosis (NUD). The lesions resolved with a prednisone taper.

NUD should be considered in patients with SLE who present with urticaria-like eruptions unresponsive to antihistamines. Early biopsy and recognition of NUD can prevent misdiagnosis and inappropriate escalation of immunosuppressive therapy.

## Introduction

Neutrophilic urticarial dermatosis (NUD) is a skin condition characterized by an eruption of erythematous macules or slightly elevated plaques that disappear within 24 to 48 hours, resembling urticaria. Histopathology reveals a dense perivascular and interstitial infiltrate of neutrophils accompanied by leukocytoclasia, with no evidence of vasculitis or dermal edema [1]. This condition was first described in 2009 by Kieffer et al., who suggested the name "neutrophilic urticarial dermatosis" because, histopathologically, this dermatosis belongs to the group of neutrophilic aseptic diseases rather than conventional urticaria [2]. The epidemiology of this condition is relatively underreported in the literature, as it is rare. Clinically, it primarily affects the trunk and extremities and may present with extracutaneous manifestations, such as fever and arthralgia [3]. NUD is commonly linked to systemic conditions, including systemic lupus erythematosus (SLE), Schnitzler syndrome, adult-onset Still's disease, cryopyrin-associated periodic syndromes, Sjögren syndrome, primary biliary cirrhosis, inflammatory bowel disease, serum sickness-like drug reactions, post-streptococcal rheumatic disease, and less commonly systemic-onset juvenile arthritis [3]. This case underscores the importance of distinguishing NUD from other urticarial disorders, particularly in patients with SLE, to avoid mismanagement.

## Case presentation

A 47-year-old woman with a long-standing history of SLE, confirmed by positive ANA, anti-dsDNA, and anti-Smith antibodies, presented with a new, mildly pruritic, burning rash of one week's duration. She reported that the individual lesions lasted one to two days before fading. Her SLE was managed with mycophenolate mofetil 1 g twice daily, prednisone 10 mg daily, and anifrolumab 300 mg IV every four weeks. She had been on this regimen for about a year.

**Figure 1:**
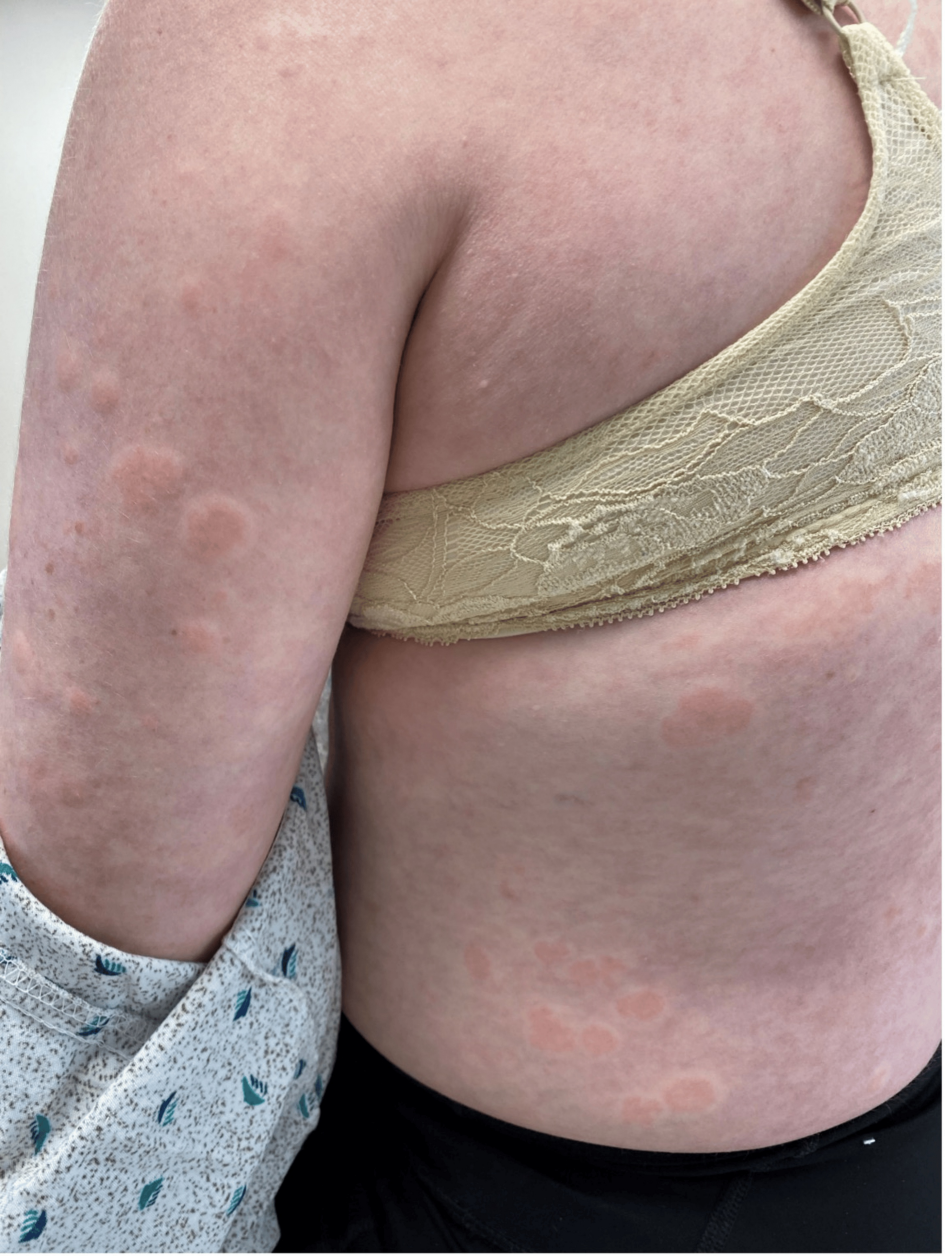
Erythematous, edematous wheals on the trunk and proximal extremities. Clinical photograph obtained with a standard digital camera under ambient clinic lighting.

On physical examination, erythematous, edematous wheals were observed on her trunk and proximal extremities (Figure 1). The lesions lacked purpura and were clinically indistinguishable from urticaria. Based on the clinical findings, she was initially treated with cetirizine 10 mg four times daily for treatment of urticaria. Despite this treatment, the rash showed minimal improvement, with persistent lesions continuing to develop.

**Figure 2:**
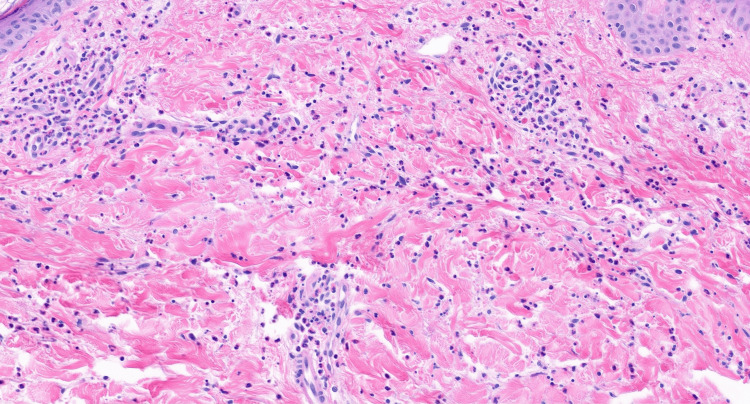
Skin biopsy showing a moderately dense interstitial and perivascular neutrophilic infiltrate with nuclear dust, and intact vessels without evidence of vasculitis or dermal edema. These findings are characteristic of neutrophilic urticarial dermatosis. Hematoxylin and eosin (H&E) stain, original magnification ×200.

The patient was initially treated with cetirizine at a high dose (40 mg daily), which did not result in clinical improvement. Due to her lack of response, history of SLE, and personal preference for further evaluation, a skin biopsy was performed. Histopathological examination revealed a moderately dense infiltrate of neutrophils and nuclear dust, with intact vessels and no evidence of vasculitis or dermal edema (Figure 2). These findings confirmed a diagnosis of NUD. She was treated with a prednisone taper (starting at 60 mg daily, gradually reduced to 40 mg and then 20 mg over 15 days), which resulted in the resolution of the lesions. Being that prednisone was initiated empirically while urticaria was still suspected, agents such as dapsone or colchicine were not prescribed. However, if the lesions persisted or returned, these steroid-sparing agents would have been considered as next-line therapy.

## Discussion

NUD is a neutrophilic dermatosis that can resemble urticaria and is linked to systemic conditions. Due to the overlap in clinical features among NUD, urticaria, and urticarial vasculitis (UV), accurate differentiation is essential for effective management. Distinguishing these conditions requires attention to systemic symptoms, associated conditions, and histopathologic findings.

Unlike urticaria, NUD is typically less itchy and does not respond to antihistamines. Histopathologically, while urticaria may occasionally show numerous neutrophils, it lacks neutrophilic dust and neutrophilic epitheliotropism, where neutrophils extend into epithelial structures [4]. Treatment for urticaria involves antihistamines, while NUD usually does not respond to them. Identifying NUD is crucial, as it necessitates evaluation for related systemic conditions.

Both NUD and UV can occur in patients with systemic conditions, like SLE, and may present with symptoms, such as fever and joint pain. However, the wheals of UV are usually more purpuric, last longer, and often resolve with post-inflammatory hyperpigmentation [1]. Histopathologically, UV is characterized by leukocytoclastic vasculitis, vessel destruction, and extravasated erythrocytes; none of which are present in NUD. Treatment for UV typically focuses on managing the underlying condition, such as controlling SLE. Unlike chronic spontaneous urticaria (CSU), which typically responds to increasing doses of antihistamines and may require biologics such as omalizumab or dupilumab in refractory cases, NUD does not resolve with antihistamines and necessitates histopathologic confirmation. This distinction highlights the value of biopsy in patients with systemic autoimmune disease when urticaria-like eruptions do not respond to antihistamines.

The findings of this case are compatible with previous reports of NUD in systemic lupus erythematosus. Gusdorf et al. conducted a retrospective study of seven patients with SLE who developed NUD [1]. The study emphasized the clinical significance of distinguishing NUD from UV to ensure proper management [1]. Additionally, Kieffer et al. first characterized NUD as a distinct neutrophilic dermatosis in 2009, shining light on its similarity to urticaria clinically despite its different histopathology [2]. In 2020, Gusdorf and Lipsker further stressed the systemic associations of NUD by noting its role as a diagnostic bridge between monogenic and polygenic autoinflammatory disorders [3].

The association between NUD and SLE is thought to result from underlying immune dysregulation in SLE, which promotes neutrophilic infiltration and inflammation in the skin via elevated cytokine activity. This results in the emergence of NUD in predisposed individuals with SLE, emphasizing the need to consider systemic autoimmune disease in patients presenting with these urticaria-like eruptions [1].

NUD is an uncommon but significant consideration in patients with SLE presenting with urticarial eruptions. Its management differs from that of SLE flares, UV, and urticaria. Recognizing NUD as a distinct clinical and histopathological entity is crucial to prevent unnecessary escalation of immunosuppressive therapies aimed at SLE. Instead, medications like dapsone or colchicine, which target neutrophil activity, have proven effective for NUD, similar to their use in other neutrophilic dermatoses [5,6]. Systemic corticosteroids may provide short-term control, particularly in patients with accompanying autoimmune diseases such as SLE, but are generally not considered first-line long-term therapy for NUD.

## Conclusions

This case highlights the importance of histopathological evaluation in guiding treatment for patients with SLE with resistant urticarial lesions. If biopsy findings suggest NUD in a patient without a definitive diagnosis, further evaluation for SLE and related systemic conditions is warranted. NUD should be considered in patients whose urticarial-like rashes do not respond to standard treatments, as misidentifying it as a lupus flare could lead to inappropriate management. Early recognition and tailored treatment of NUD can significantly improve patient outcomes, emphasizing the need for awareness of this distinct condition among dermatologists and rheumatologists.
